# Nanomedicine for drug delivery in South Africa: a protocol for systematic review

**DOI:** 10.1186/s13643-018-0823-5

**Published:** 2018-10-06

**Authors:** Trust Saidi, Jill Fortuin, Tania S Douglas

**Affiliations:** Division of Biomedical Engineering, Department of Human Biology, Faculty of Health Sciences, University of Cape Town, Anzio Road, Observatory, Cape Town, 7925 South Africa

**Keywords:** Nano, Nanomedicine, Nanotechnology, Drug delivery, Diseases

## Abstract

**Background:**

The emergence of nanomedicine in the past decade has changed the landscape of disease diagnosis and treatment. Nanomedicine makes use of nanostructures for applications in different fields of medicine, including drug delivery, biosensors, neuro-electronic interfaces, in vivo imaging, and cell-specific molecular interactions. Despite its relative infancy, nanomedicine has generated a significant body of research as evidenced by peer reviewed literature and several patents. This proposed systematic review will focus specifically on drug delivery systems in which nanoparticles are used to enhance the pharmacological and therapeutic properties of drugs. The strength of nanoparticulate drug delivery systems is their ability to alter the pharmacokinetics and bio-distribution of drugs.

Globally, the discourse on nanomedicine is dominated by research being done in the developed countries of Europe and in the United States of America. Less attention has been given to the applications of nanomedicine in developing countries, particularly Africa. There is dearth of information on the applications of nanomedicine in terms of drug delivery with particular reference to which diseases are being targeted generally in Africa. The review will describe the specific diseases that are being targeted and the progress being made in South Africa, with a view to determining whether the applications of nanomedicine are being appropriated to address the context-specific challenges in this country or if they mimic what is being done globally.

**Methods:**

Keywords related to nanomedicine and drug delivery will be combined to build a search strategy for each of the following databases: PubMed, Cochrane Library (including Cochrane Central Register of Controlled Trials (CENTRAL), Cochrane Database of Systematic Reviews, Cochrane Methodology Register), Google Scholar, NHS Health Technology Assessment Database and Web of Science. We will also check reference lists of included studies for other eligible reports and search unpublished data. To ensure that the search is comprehensive, grey literature will be searched extensively. Literature to be included will have nanomedicine in drug delivery as the primary application and report on the specific diseases that are targeted in South Africa. Two authors will independently screen the search output, select studies and extract data; discrepancies will be resolved by consensus and discussion. When no consensus is reached, the third author will be consulted

**Discussion:**

The systematic review will inform the government, policy-makers, investors, health professionals, scientists, and engineers about the applications of nanomedicine in drug delivery. In particular, it will identify the diseases targeted by the application of nanomedicine for drug delivery and the progress being made in South Africa as the disease burden of this country differs from that of developed countries where nanomedicine has been widely used for drug delivery.

**Systematic review registration:**

PROSPERO CRD42017057388

**Electronic supplementary material:**

The online version of this article (10.1186/s13643-018-0823-5) contains supplementary material, which is available to authorized users.

## Background

Nanomedicine is defined in a variety of ways, but an all-encompassing definition includes the development of nanoparticles, nanostructured surfaces and nanoanalytical techniques for molecular diagnostics, treatment, follow-up and therapy of diseases [1]. Research into the delivery and targeting of pharmaceutical, therapeutic and diagnostic agents is at the forefront of nanomedicine [2]. This emerging field brings current advances in chemistry, physics, biology and materials science to bear on diagnosis and therapy for a wide range of diseases [3].

The nano-scaled particles used in nanomedicine have revolutionised drug delivery, allowing therapeutic agents to be selectively targeted on organ, tissue and cell-specific levels, while minimising exposure of healthy tissues to drugs [4]. The use of nanoparticles for drug delivery is meant to overcome the inherent limitations of bio-macromolecular therapeutics, which include a short plasma half-life, poor stability and potential immunogenicity [5, 6]. Although conventional drug delivery systems are effective at releasing drugs in a controlled fashion to produce a high local concentration, their scope is limited to targeting tissues rather than individual cells [6].

The ability of nanoparticles to interact with cells and tissues at a molecular level provides them with a distinct advantage over other polymeric or macromolecular substances [7]. Nanoparticles are attractive for medical purposes due to their unique features, such as their surface to mass ratio that is much larger than that of other particles, their quantum properties and their ability to adsorb and carry other compounds [8, 9]. Due to the advantage of their size, nanoparticles have been shown to be robust drug delivery systems and are useful for encapsulating drugs and enabling more precise targeting with a controlled release, especially for drugs that have poor solubility and absorption [10–12]. Some of the applications of nanoparticles for drug delivery have already reached clinical practice, such as liposomal doxorubicin used to treat different forms of cancer, or liposomal amphotericin B, which treats fungal infections often associated with aggressive anti-cancer treatment [13]. Furthermore, one of the positive outcomes of nanomedicine involves the use of nanoparticles to deliver drugs through the blood-brain barrier for targeting brain tumours [14].

New types of intelligent nanoparticles respond to an externally applied field, be it magnetic, focused heat, or light, in ways that make them ideal therapeutics or therapeutic delivery vehicles [15]. The major drive is to facilitate the identification of precise targets (cells and receptors) related to specific clinical conditions and to use the appropriate nanocarriers to achieve the required responses, while minimising the side effects [16]**.** This can make in vivo delivery of drugs that pose serious delivery problems, a relatively easy task [17–19]. Drug release and bio-degradation are important parameters for developing successful formulations that differ from conventional therapeutic agents; the latter are distributed non-specifically in the body where they affect both diseased and normal cells, thereby limiting the dose achievable within the diseased cells and also resulting in sub-optimal treatment due to excessive toxicities [20].

Developed countries have taken a leading role in the field of nanomedicine as they are investing large amounts of money into developments in the technology [21]. For instance, many academic groups in Europe, Japan and the United States of America are leading nanomedicine research on delivery systems and clinical development of nanodrugs [22]. In Africa, nanomedicine is an emerging field, compared to the rest of the world, and the limiting factor for research is the cost of the required equipment [23]. South Africa, which is a leading country in terms of health care services and biomedical research in Africa, is one of the countries engaged in nanomedicine research and product development on the continent [24], hence the focus of this study. The country produces the highest scientific output per capita on the continent [25]. The purpose of the systematic review is to identify the diseases for which nanomedicine is used for drug delivery in South Africa and describe the progress made on the disease areas. This is motivated by the fact that the applications of nanomedicine tend to differ across countries or regions depending on the disease burden. A study on South Africa will begin to reveal the focus areas of nanomedicine for drug delivery in developing countries.

## Methods and systematic review design

### Study design and scope

We will conduct a systematic review of studies pertaining to nanomedicine for drug delivery conducted by researchers in South Africa, with a focus on laboratory studies. This focus is motivated by the fact that nanomedicine is an emerging and specialised field in South Africa and much work is limited to fundamental laboratory-based research. All studies on nanomedicine for targeted drug delivery in South Africa will be eligible for inclusion. Primarily, nanomedicine studies include research from academic institutions, hospitals, research organisations, and pharmaceutical companies [26]. The study will include only research with an author affiliation to an institution in South Africa.

### Review questions

The systematic review proposes to address the following research questions:

- Which diseases are being targeted for drug delivery using nanomedicine in South Africa?
- What methods of drug delivery using nanomedicine are being applied for the identified diseases?
- What progress has been made on the use of nanomedicine for drug delivery with reference to the identified disease areas?

### Review objectives

In line with the study questions above, the review seeks to

1. Document the diseases which are being targeted for drug delivery using nanomedicine in South Africa by
  - Identifying the applications of nanomedicine for drug delivery in South Africa.
  - Synthesising information on the targeted diseases and the affiliations of the researchers in South Africa.
  - Critically appraising the disease profiles of studies forming the evidence base for the applications of nanomedicine in drug delivery in South Africa.
2. Provide a structured assessment of the progress of nanomedicine on drug delivery for the identified diseases in South Africa by
  - Extracting quotes from included studies on the progress of the nanomedicine for drug delivery in South Africa.
  - Coding, thematically grouping and iteratively analysing the data to characterise the progress in the use of nanomedicine for drug delivery.

### Participants/population

The population for this study consists of potential patients who could be recipients of drug delivery using nanomedicine. As nanomedicine is confined to laboratory studies as revealed by the preliminary literature search, and the focus of the proposed review is laboratory studies, included studies will not include real patients who will have been treated using the technology in South Africa. However, it is possible to infer the potential patients since nanomedicine studies target particular diseases and groups of people. For example, scientists at the Council for Scientific and Industrial Research in South Africa focus on encapsulating conventional tuberculosis drugs with nanoparticles to ensure targeted delivery for tuberculosis treatment. We can deduce from this example that the potential population consists of tuberculosis patients. This study, by virtue of being focused on the different applications of nanomedicine for drug delivery, will yield a heterogenous population consisting of patients with various disease profiles.

### Intervention

Nanomedicine interventions to be included in the study should aim at drug delivery in South Africa for different diseases. The following nano-enabled drug delivery interventions and platforms will be considered for inclusion: liposome, polymeric drug, drug polymer conjugate, protein polymer conjugate, pegylated protein, nanoparticle, nanocapsule, nano-suspension, nanocrystal, gold nanoparticle, colloidal gold, silicate nanoparticle, calcium nanoparticle, biosilic, titanium dioxide nanoparticle, solid lipid nanoparticle, fullerene drug, dendrimer drug, nanoshell, phototherapy, hypothermal therapy and magnetic nanoparticle.

### Comparison/control

Since nanomedicine is being applied to increase efficacy, safety, sensitivity and personalisation with the goal of improved therapy, the control for the study are traditional methods of drug delivery. Initial searches of literature on the applications of nanomedicine for drug delivery have shown that by default, use of nanomedicine for drug delivery is compared with existing practices.

### Outcomes

The outcomes of the systematic review will focus on frequency of publication and themes studied across retrieved literature. The diseases being targeted using nanomedicine for drug delivery in South Africa, the progress in nanomedicine for drug delivery based on thematic analysis and the contributing institutions will be presented, to describe the nanomedicine ecosystem in South Africa. The relationship between possible drug delivery platforms and diseases will be depicted in a map.

### Search strategy

We will identify relevant studies within the period starting in 1 January 2006 to date with language of publication limited to English. The start date has been selected as 1 January 2006, as this is the time when the South African nanotechnology strategy and an associated 10-year plan for nanotechnology were implemented. The databases to be searched include PubMed (NLM); Scopus; PsycINFO, EMBASE; Cochrane Library (Wiley) (including Cochrane Central Register of Controlled Trials (CENTRAL), Cochrane Database of Systematic Reviews, Cochrane Methodology Register, NHS Health Technology Assessment Database and Web of Science (Thomson Reuters). The studies to be included will be selected using predefined search terms adapted for the databases to be used. The terms comprise both free text word and medical subject heading (MeSH). We will use a Boolean search string, which includes keywords, namely, nanomedicine, drug delivery and South Africa. PubMed will be used as the primary database by virtue of being one of the largest archives of biomedical literature, and it conducts a review of the scientific quality of journals before indexing [24]. The preliminary search on PubMed is presented in Table 1, and it will be replicated for the other databases.

**Table 1:** Search strings for extracted literature

Population		
#1	MeSH terms:	Drug Delivery Systems
#2	All Fields:	drug delivery OR targeted drug OR drug targeting
#3	#1 OR #2	
Intervention		
#4	MeSH terms:	Nanotechnology OR Nanostructures
#5	All fields:	Nanomedicine OR Nanotechnology
#6	#4 OR #5	
Outcome		
#7	MeSH terms:	South Africa
#8	Free text:	South Africa OR South African
#9	#7 OR #8	
#10	#3 AND #6 AND #9	January 2006 to April 2018
#11	Filter #10 by specifying the time period	

### Other sources

Nanomedicine is a specialised topic and as a result, to ensure that the search is comprehensive, the grey literature will be searched extensively. This includes searching relevant conference proceedings, the websites of scientific societies and pharmaceutical companies, the New York Academy of Literature and Open Grey. The reference lists of appropriate studies will be assessed. Full text articles of the studies extracted from the reference list will be obtained and reviewed for additional information. Unpublished studies will be identified, and the same eligibility criteria will be applied to select relevant studies.

### Study selection

**Fig. 1:**
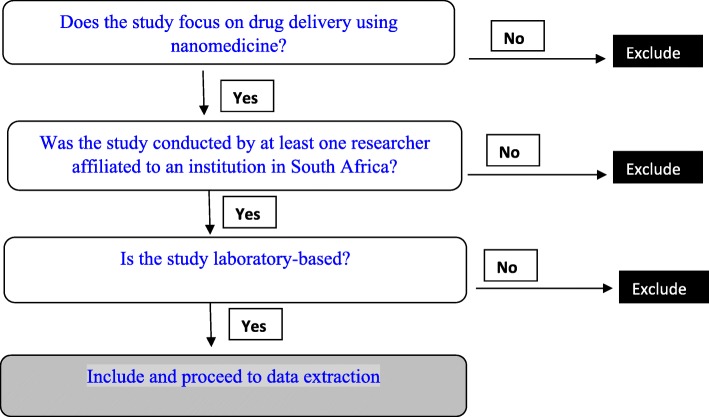
Selection criteria applied to abstracts retrieved via literature search

Once the search strategy has been developed and tested, the first author will retrieve all the relevant articles from the various databases. All the literature obtained will be saved in Endnote reference management software for further analysis. The titles and abstracts of studies identified in the literature search will be screened independently by two authors for eligibility. Prospective studies and studies retrospectively focusing on the applications of nanomedicine for drug delivery will be included. We acknowledge that nanomedicine is relatively new in South Africa, and we will focus on laboratory studies. The final assessment for inclusion will be made by consensus, based on the full text of articles. Discrepancies and disagreements will be resolved by a third author overseeing the review process. Clear reasons for exclusion will be documented by each reviewer. Figure 1 outlines the study selection procedures employed.

### Data extraction

A standardised form has been developed and will be piloted and revised if necessary to extract data from the eligible studies. Full texts of included abstracts will be retrieved and data extracted as per the pre-specified template (Additional file 1). Key information to be extracted includes:

- Author/s and year of the study
- Type of facility/environment
- Affiliation of author
- Type of participant/study population/demographic characteristics
- Type of nanomedicine drug delivery intervention
- Type of targeted disease
- Type of study (i.e. study design)
- Type of outcome measured
- Findings/results

Data will be entered into Review Manager (RevMan) software, Version 5.3. (Copenhagen: The Nordic Cochrane Centre, the Cochrane Collaboration, 2011). The first and second authors will verify the data entered, for missing or incorrect data. When deemed necessary, the authors of studies will be contacted for missing or incomplete data. Should there be no response from the authors, the limited data available will be included and the implications of missing/incomplete data will be discussed.

### Data analysis and synthesis

**Fig. 2:**
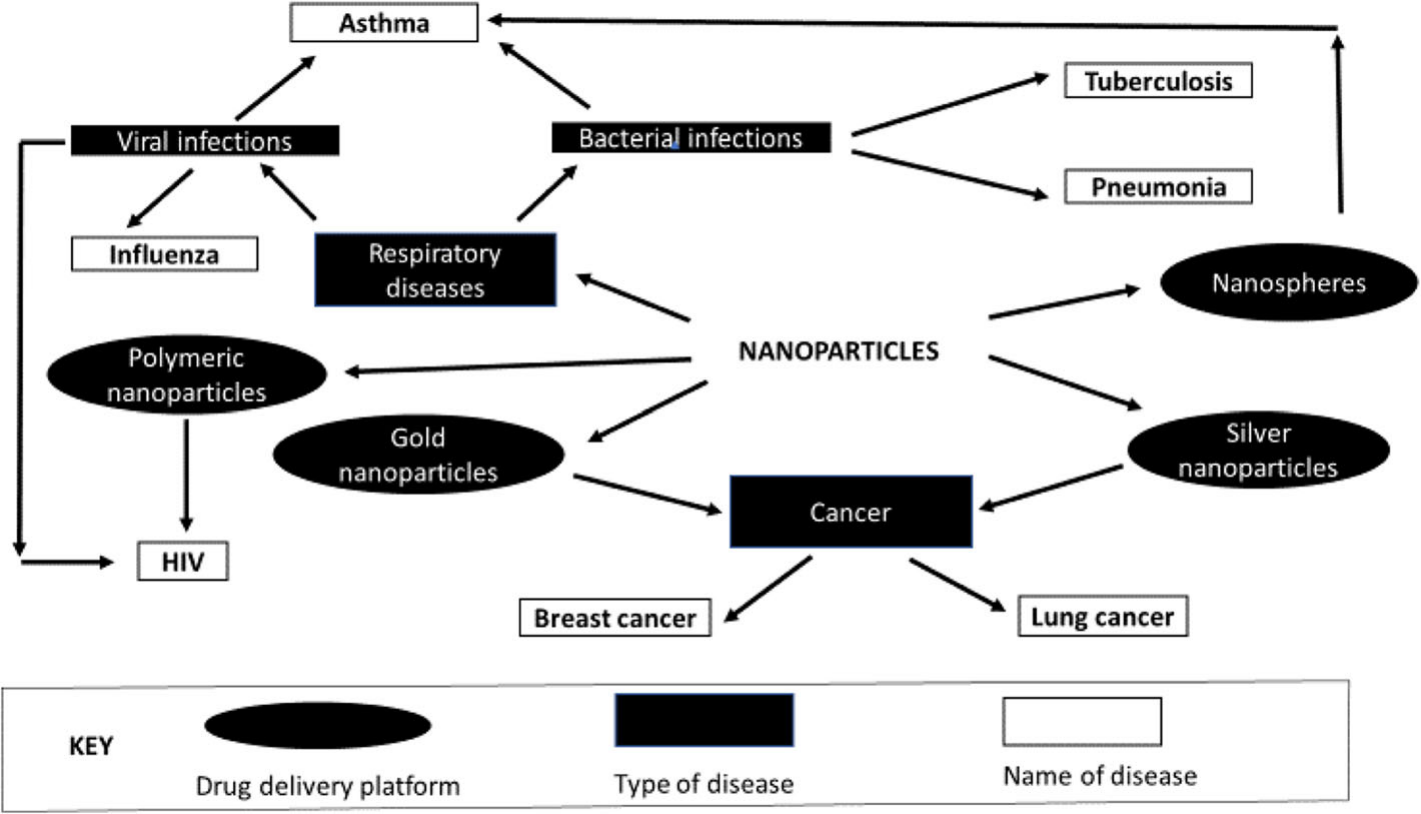
Example of nanomedicine applications depicting the platforms for drug delivery and diseases being targeted in South Africa [29–31]

The extracted data will be presented in an evidence table organised by targeted diseases, methods used for drug delivery, institutions and/or stakeholders involved, and milestones achieved in nanomedicine. We will review the use and progress of nanomedicine for drug delivery in South Africa by developing descriptive themes to answer each research question and synthesise extracted data through a narrative synthesis [27]. The heterogeneity of the studies reviewed will be explored through narrative synthesis. As per Popay et al. [28], we will synthesise insights derived from the reviewed literature in order to provide a nuanced description of how nanomedicine is being applied for drug delivery in South Africa, including an account of the methods of drug delivery and the progress made. Two reviewers will code the extracted data independently and conduct a thematic analysis to determine the patterns that are associated with the research questions. The findings will be mapped to create a holistic picture of the landscape of nanomedicine for drug delivery in South Africa. Studies with the same or related outcomes, study setting (e.g. university, science council, non-governmental organisation), target disease (e.g. HIV, TB, cancer), drug delivery (e.g. liposome, nanoparticle, drug polymer conjugate) or type of study (e.g. laboratory study) will be pooled to facilitate analysis of the data. Figure 2 shows an example of data synthesis; it portrays the application of nanoparticles, the drug delivery platform used, and the type of disease [29–31]. Figure 2 shows that cancer is targeted by gold and silver nanoparticles [30]. Breast and lung cancer are the types of cancers that are targeted. Polymeric nanoparticles are drug delivery platform that is also used in HIV [31], whereas nanospheres target asthma, which is a respiratory disease [29].

The preliminary sketch depicted in Fig. 2 on nanomedicine and diseases being targeted in South Africa will be expanded to include the institutions involved and how they are connected. The progress in the use of nanomedicine on the targeted diseases will be analysed in relation to conventional treatment.

## Discussion

The surge of applications of nanotechnology in developed countries has raised questions, with some scholars arguing that inequitable distribution of the benefits and profits will occur and accumulate in the north [32]. Nanomedicine, a recent offshoot of nanotechnology, is assumed to follow the same trajectory. Since 2005, South Africa has invested in research on nanomedicine focusing on pro-poor initiatives which prioritise diseases such as HIV/AIDS, malaria and tuberculosis. Considering the human, financial, and physical resources that the country has invested in nanomedicine, it is time to reflect on how the research is progressing. The proposed systematic review will inform further research and innovation in terms of research activities in South Africa in the field of nanomedicine.

We acknowledge the limitation that our review is restricted to laboratory studies as clinical trial results are not yet available. Since there are few laboratories in South Africa that are designed to handle research on materials at nanoscale, we acknowledge that we may not be able to conduct a meta-analysis due to limited research activity. The initial scoping exercises which we conducted revealed that there are few, but dominant, research groups that engage in nanomedicine and it is more likely that the review will be limited to a few South African institutions and researchers. In addition, the reliance on published data may predispose the review to publication bias.

Despite the limitations mentioned above, the proposed systematic review is of value both theoretically and practically. The study aims to provide an up to date account of the application of nanomedicine for drug delivery in South Africa. We seek to identify the diseases targeted for drug delivery using nanomedicine and synthesise information not only on the methods of intervention but also the progress made so far. The review will be essential to health professionals, policy-makers, funding organisations and the public at large in establishing the extent to which South Africa, a leading country in providing health care services and conducting biomedical research in sub-Saharan Africa, is exploiting the new opportunities provided by nanomedicine for the benefit of its health system.

### Report

This systematic protocol has been registered with PROSPERO, the international prospective register of systematic reviews. The registration number is CRD42017057388. The authors acknowledge that it adheres to the PRISMA-P 2015 checklist as a condition for submission of systematic review protocols (see Additional file 2). The review will follow the recommendations of the Cochrane handbook for systematic reviews of interventions for the design and analysis plan development and the preferred reporting items for systematic reviews and meta-analyses (PRISMA) will be followed in presenting the results.

## Additional files

Additional file 1: Data extraction form. (DOC 135 kb)

Additional file 2: PRISMA-P 2015 checklist. (DOCX 31 kb)

## Abbreviations

CENTRAL: Cochrane Central Register of Controlled Trials
NHS: National Health Service
PRISMA: Preferred Reporting Items for Systematic Reviews and Meta-Analyses
