# Supplementary material for: Nanomedicine for drug delivery in South Africa: a protocol for systematic review
Source: Syst Rev. 2018 Oct 6;7:154. doi: 10.1186/s13643-018-0823-5 (PMC6173875; doi:10.1186/s13643-018-0823-5)
Supplement: Supplementary file 1 — Data extraction form. (DOC 135 kb) [file 13643_2018_823_MOESM1_ESM.doc]

# Data Extraction Form

Nanomedicine for Drug Delivery in South Africa

**Adapted from:**  <http://bmjopen.bmj.com/content/bmjopen/7/6/e015626/DC1/embed/inline-supplementary-material-1.pdf?download=true>

| Review title or ID |  |
| --- | --- |
| Study ID *(surname of first author and year first full report of study was published e.g. Smith 2001)* |  |

# General Information

| Date form completed *(dd/mm/yyyy)* |  |
| --- | --- |
| Name/ID of person extracting data |  |
| Reference citation |  |
| Study author contact details |  |
| Publication type  *(e.g. full report, abstract, letter)* |  |

# Study eligibility

| Study Characteristics | Eligibility criteria  *(Insert inclusion criteria for each characteristic as defined in the Protocol)* | | Eligibility criteria met? | | | Location in text or source *(pg & ¶/fig/table/other)* |
| --- | --- | --- | --- | --- | --- | --- |
|  |  |  | Yes | No | Unclear |  |
| Type of study | No limitation on the type of studies | |  |  |  |  |
|  |  |  |  |  |  |  |
| Participants | Potential patients who could be recipients of drug delivery using nanomedicine. | |  |  |  |  |
| Types of intervention  Nanomedicine | Drug delivery methods of nanomedicine such as liposome, polymeric drug, drug polymer conjugate, protein polymer conjugate and pegylated protein. | |  |  |  |  |
| Types of comparison | Nanomedicine will be compared with conventional treatment. | |  |  |  |  |
| Types of outcome measures | Frequency of publication, themes studied across retrieved literature, list of diseases being targeted by nanomedicine in South Africa and the progress | |  |  |  |  |
| INCLUDE | | EXCLUDE | | | | |
| Reason for exclusion |  | | | | | |
| Notes: | | | | | | |

**DO NOT PROCEED IF STUDY EXCLUDED FROM REVIEW**

Characteristics of included studies

## Methods

|  | **Descriptions as stated in report/paper** | | **Location in text or source** *(pg & ¶/fig/table/other)* |
| --- | --- | --- | --- |
| **Aim of study** *(e.g. efficacy, equivalence, pragmatic)* |  | |  |
| **Design***(e.g. parallel, crossover, non-RCT)* |  | |  |
| **Unit of allocation**  *(by individuals, cluster/ groups or body parts)* |  | |  |
| **Start date** |  | |  |
| **End date** |  | |  |
| **Duration of participation***(from recruitment to last follow-up)* |  | |  |
| **Ethical approval needed/ obtained for study** | Yes No Unclear |  |  |

## Participants

|  | Description | | Location in text or source *(pg & ¶/fig/table/other)* |
| --- | --- | --- | --- |
| Population description *(from which study participants are drawn)* |  | |  |
| Setting*(including location and social context)* |  | |  |
| Method of recruitment of participants *(e.g. phone, mail, clinic patients)* |  | |  |
| Informed consent obtained | Yes No Unclear |  |  |

## Outcomes

|  | Description as stated in report/paper | Location in text or source *(pg & ¶/fig/table/other)* |
| --- | --- | --- |
| Outcome name |  |  |
| Time points measured *(specify whether from start or end of intervention)* |  |  |
| Time points reported |  |  |
| Outcome definition *(with diagnostic criteria if relevant)* |  |  |
| Person measuring/ reporting |  |  |
| Unit of measurement |  |  |

## Other

| **Study funding sources**  *(including role of funders)* |  |  |
| --- | --- | --- |
| **Possible conflicts of interest**  *(for study authors)* |  |  |

# Risk of Bias assessment

| Domain | Risk of bias | | | Support for judgement  *(include direct quotes where available with explanatory comments)* | Location in text or source *(pg & ¶/fig/table/other)* |
| --- | --- | --- | --- | --- | --- |
|  | Low | High | Unclear |  |  |
| Random sequence generation  *(selection bias)* |  |  |  |  |  |
| Allocation concealment  *(selection bias)* |  |  |  |  |  |
| Blinding of participants and personnel  *(performance bias)* |  |  |  | Outcome group: All/ |  |
| Blinding of outcome assessment  *(detection bias)* |  |  |  | Outcome group: All/ |  |

# Data and analysis

| Results | Intervention | | Comparison | |  |
| --- | --- | --- | --- | --- | --- |
|  | No. with event | Total in group | No. with event | Total in group |  |
|  |  |  |  |  |  |
| Any other results reported *(e.g. odds ratio, risk difference, CI or P value)* |  | | | |  |
| No. missing participants |  | |  | |  |
| Reasons missing |  | |  | |  |
| No. participants moved from other group |  | |  | |  |
| Reasons moved |  | |  | |  |
| Unit of analysis *(by individuals, cluster/groups or body parts)* |  | | | |  |

# Other information

|  | **Description as stated in report/paper** | **Location in text or source** *(pg & ¶/fig/table/other)* |
| --- | --- | --- |
| **Key conclusions of study authors** |  |  |
| **References to other relevant studies** |  |  |
| **Correspondence required for further study information** *(from whom, what and when)* |  | |
